# Common isolation of New Delhi metallo-beta-lactamase 1-producing Enterobacteriaceae in a large surgical hospital in Vietnam

**DOI:** 10.1007/s10096-015-2345-6

**Published:** 2015-03-03

**Authors:** H. H. Tran, S. Ehsani, K. Shibayama, M. Matsui, S. Suzuki, M. B. Nguyen, D. N. Tran, V. P. Tran, D. L. Tran, H. T. Nguyen, D. A. Dang, H. S. Trinh, T. H. Nguyen, H. F. L. Wertheim

**Affiliations:** 1 National Institute of Hygiene and Epidemiology, Yersin Street 1, Hanoi, Vietnam; 2 Pasteur Institute, Paris, France; 3 National Institute of Infectious Diseases, Tokyo, Japan; 4 Viet-Duc Hospital, Hanoi, Vietnam; 5 Wellcome Trust Major Overseas Program, Oxford University Clinical Research Unit, Hanoi, Vietnam; 6 Nuffield Department of Clinical Medicine, Centre for Tropical Medicine, University of Oxford, Oxford, UK

## Abstract

This study sought to monitor the presence of carbapenem-resistant Enterobacteriaceae (CRE) and the proportion New Delhi metallo-beta-lactamase 1 (NDM-1)-producing bacteria between August 2010 and December 2012 in a surgical hospital in Vietnam. We identified 47 CRE strains from a total of 4,096 Enterobacteriaceae isolates (1.1 %) that were NDM-1-positive from 45 patients admitted to 11 different departments, with the majority being from the urology department. The NDM-1 gene was found in seven different species. Genotyping revealed limited clonality of NDM-1-positive isolates. Most of the isolates carried the NDM-1 gene on a plasmid and 17.8 % (8/45) of those were readily transferable. We found five patients at admission and one patient at discharge with NDM-1-positive bacteria in their stool. From 200 screening environmental hospital samples, five were confirmed to be NDM-1-positive and included *Acinetobacter* species (*n* = 3) and *Enterobacter aerogenes* (*n* = 2). The results reveal that NDM-1-producing Enterobacteriaceae are commonly isolated in patients admitted to a Vietnamese surgical hospital and are also detected in the hospital environment.

## Introduction

Garrett Hardin’s influential 1968 essay in Science launched the idea that common goods are under threat when individuals use a shared resource in an unsustainable way, also known as the “tragedy of the commons.” [1]. The tragedy is well known to affect global issues, from climate change to biodiversity conservation. The shared, and originally natural, resource of antimicrobials is another example of how a resource, antibiotic effectiveness, on which we all depend can be destroyed by using antibiotics injudiciously [2]. The problem of antibiotic resistance has reached another climax with the discovery of NDM-1, New Delhi metallo-beta-lactamase 1 (MBL), a gene that confers resistance to the last-resort antibiotic class of carbapenems [3]. The NDM-1 gene encodes an enzyme that hydrolyses and inactivates carbapenems. Sequence comparisons show that the NDM-1 gene is a chimera, formed by in-frame fusion of a pre-existing MBL gene with the aminoglycoside resistance gene *aphA6* [4]. The resistance gene is usually located on a plasmid that can be easily transferred to other bacterial strains via horizontal gene transfer. NDM-1 has already been identified on a variety of plasmids and in a large variety of Gram-negative pathogens [5, 6]. In addition, sequencing data of plasmids containing the NDM-1 gene reveal the existence of additional resistance genes [6, 7]. A bacterium carrying such a plasmid is usually resistant to all antibiotics, apart from tigecycline and colistin [6, 8].

Through increasing immigration, travel and environmental spread, NDM-1 is reported from most continents around the world, including more and more reports from South East Asia [5]. In Vietnam, antibiotic resistance rates have been reportedly high. A combination of a high infectious diseases burden, unrestricted access to antibiotics and poor infection control measures contribute to the emergence of antibiotic resistance in Vietnam [9, 10].

NDM-1 has already been reported in Vietnamese patients, healthy volunteers and the environment [11–13]. However, the magnitude of the problem of carbapenem resistance encoded by NDM-1 remains unknown. We, therefore, carried out a surveillance project to detect carbapenem-resistant bacteria containing the NDM-1 gene in a large surgical hospital in Vietnam and collected key epidemiological data.

## Materials and methods

### Site

This study was conducted over a period of 28 months from August 2010 to December 2012 in a large surgical hospital in Hanoi, Vietnam. Molecular and microbiological analyses were performed in the hospital clinical microbiology laboratory and at the National Institute of Hygiene and Epidemiology (NIHE) in Hanoi.

### Study participants and sample collection

Enterobacteriaceae isolated from clinical specimens from any hospital department were tested for antibiotic susceptibility using disc diffusion testing according to international guidelines [14]. The microbiology consultants of the hospital were requested to collect and send all of bacteria strains resistant at least to one of antibiotics in the carbapenem group to the NIHE for further identification and NDM-1 confirmatory testing by polymerase chain reaction (PCR) and sequencing.

Demographic and basic clinical information were collected from patients with carbapenem-resistant bacteria: age, gender, date of admission, clinical department, clinical diagnosis, origin of collected sample, isolated bacterial strains and date of sample collection. Treatment and clinical outcome data were not available for this study.

### Microbiology

Bacterial species identification was performed using commercial biochemical testing kits (API20E and API20NE, bioMérieux, France). The minimum inhibitory concentrations (MICs) for carbapenem resistance were obtained by agar dilution according to the Clinical and Laboratory Standards Institute (CLSI) 2012 guidelines and the European Committee on Antimicrobial Susceptibility Testing (EUCAST) breakpoint for colistin [15].

The MBL Etest (bioMérieux, France) was used to screen for metallo-beta-lactamase production. The NDM-1 gene of bacterial isolates was detected by PCR using specific primers (Kp-ndm1-F: 5′-ttcgacccagccattggcggcga-3′ and Kp-ndm1-R: 5′-atgcacccggtcgcgaagctgag-3′), as described previously [3]. We also performed PCRs to detect genes encoding extended-spectrum beta-lactamases (ESBLs; TEM, SHV and CTX-M). Furthermore, we checked for other genes encoding for carbapenemases (KPC, IMP, VIM, SIM) and OXA-48, as described elsewhere [16–21]. Positive resistant gene PCR products were sequenced for confirmation.

Conjugational transfer of NDM-1 plasmids to the laboratory strain *Escherichia coli* J53 was done on LB broth without selection. After 16 h, the mixed cultured was centrifuged, suspended in saline and plated onto MacConkey agar containing sodium azide (100 mg/L) and meropenem (2 mg/L). Transconjugants were confirmed to have NDM-1 by PCR analysis. Plasmids were subsequently isolated and typed as described by Carattoli and colleagues [22].

NDM-1-positive bacterial isolates were genotyped by pulsed-field gel electrophoresis (PFGE), using XbaI restriction enzyme (Roche Diagnostic, Mannheim, Germany) to digest the bacterial genomes in agarose blocks. The resulting DNA fragments were separated by PFGE on CHEF-DR III apparatus (Bio-Rad, Hercules, CA, USA) for 20 h at 6 V/cm at 14 °C with an initial pulse time of 0.5 s and a final pulse time of 30 s, as described elsewhere [23].

For the characterisation of plasmids carrying the NDM-1 gene, we treated genomic DNA in agarose blocks with restriction enzyme S1 (Invitrogen, Abingdon, UK) and separated them by PFGE. The Biometra system (Analytik Jena, Germany) was used to transfer DNA fragments from the gel to nylon membrane and they were hybridised with NDM-1 probe labelled in an HL-2000 HybriLinker (UPV, Germany). Hybridisation plasmids with NDM-1 were cut from the gel, purified and typed as described by Carattoli and colleagues [22].

### Rectal carriage of NDM-1 at admission and discharge

We assessed the carriage rate of NDM-1-positive bacteria in patients at admission and discharge among 103 randomly selected patients admitted to the urology department, which was the department where most of the NDM-1-positive patients were detected. Between May 2012 and October 2012, we screened up to five patients per day by collecting rectal swabs within 24 h after admission and a second rectal swab just before discharge. We aimed to enroll 10–15 patients per week. Rectal swabs were collected using sterile cotton-tipped swabs (Copan, Italy) and inoculated on 5 ml of LB broth containing imipenem (2 mg/L) for 6–8 h at 37 °C and then plated onto selective MacConkey agar plates supplemented with 2 mg/L imipenem and incubated at 37 °C. After 24–48 h, five randomly selected single colonies were collected and tested for susceptibility to carbapenems and the presence of the NDM-1 gene, as detailed above.

### NDM-1 in the hospital environment

During the period of one week, 200 environmental samples in the hospital were collected and tested for carbapenem-resistant bacteria. Samples were collected from the patient’s bed, medical and non-medical waste, floor, toilets, medical trolley, office table, nurse’s telephone and vacuum system for suctioning. Sterile cotton-tipped swabs (Copan, Italy) were used to swab the surface of the collection sites and inoculated on 5 ml of LB broth containing imipenem (2 mg/L) for 6–8 h at 37 °C and then plated onto selective MacConkey agar plates supplemented with 2 mg/L imipenem and incubated at 37 °C. After 24–48 h, five randomly single colonies were selected and tested for susceptibility to carbapenems and the presence of the NDM-1 gene, as detailed above.

### Statistical methods

We used the total number of collected isolates as the denominator for the carbapenem resistance positivity rate calculations by SPSS, 20.0. PFGE data were analysed using BioNumerics version 6.5 software (BioNumerics, USA).

### Ethics statement

Ethical approval was obtained from the Ethical Committee of the NIHE in 2010. Patients in the rectal carriage study provided informed consent to participate in the study.

## Results

**Fig. 1:**
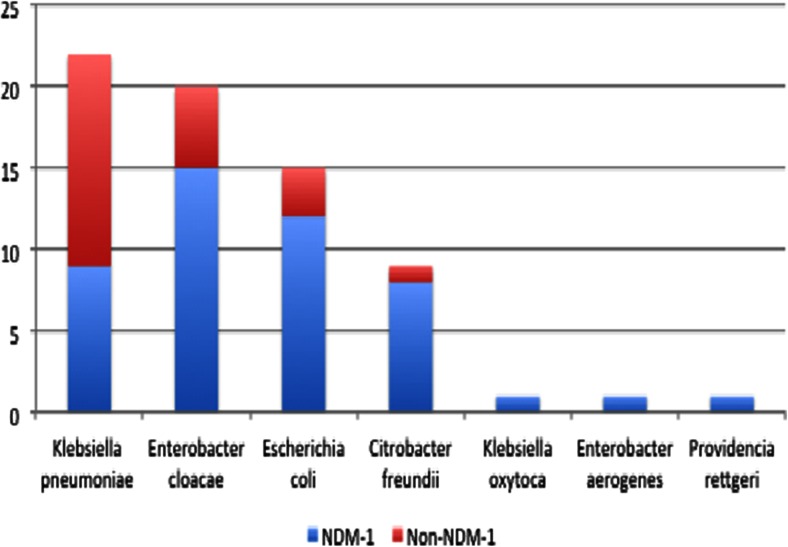
Proportion of NDM-1 among phenotypic of carbapenem-resistant bacteria

We identified 69 CRE, which included the following seven species: *Klebsiella pneumoniae* (*n* = 22), *Enterobacter cloacae* (*n* = 20), *E. coli* (*n* = 15), *Citrobacter freundii* (*n* = 9), *K. oxytoca* (*n* = 1), *E. aerogenes* (*n* = 1) and *Providencia rettgeri* (*n* = 1). These were isolated from a total of 4,096 Enterobacteriaceae analysed between August 2010 and December 2012. Of these 69 CRE, 47 (68.1 %) were NDM-1-positive and belonged to seven different bacterial species: *E. cloacae* (*n* = 15; 32 %), *E. coli* (*n* = 12; 26 %), *K. pneumoniae* (*n* = 9; 19 %), *C. freundii* (*n* = 8; 17.%), *P. rettgeri* (*n* = 1), *E. aerogenes* (*n* = 1) and *K. oxytoca* (*n* = 1) (Fig. 1). The most common specimen among the NDM-1-positive isolates was urine (55.5 %), followed by tracheal aspirates (37.8 %), bronchial secretions and blood (6.7 %). The 47 NDM-1-positive strains were isolated from 45 patients. Two patients had two different bacterial NDM-1-positive species in their urine: one patient had *C. freundii* and *E. cloacae* and the other patient had *E. cloacae* and *P. rettgeri*.

The median patient age was 39.5 years (range: 16 to 95 years). Among the NDM-1-positive patients, the male to female ratio was 10.3. None of the patients had a travel history to India or Pakistan, which are considered NDM-1 hotspots. Twenty-six NDM-1-infected patients (53.3 %) were admitted to the hospital for reasons related to urinary tract diseases. All of the NDM-1-positive isolates were a cause of either a hospital-acquired infection or colonisation. One death occurred in a trauma patient (cervical fracture), which was attributed to septic shock from bloodstream infection with an NDM-1-positive isolate. All other NDM-1-positive patients were discharged alive from the hospital.

**Fig. 2:**
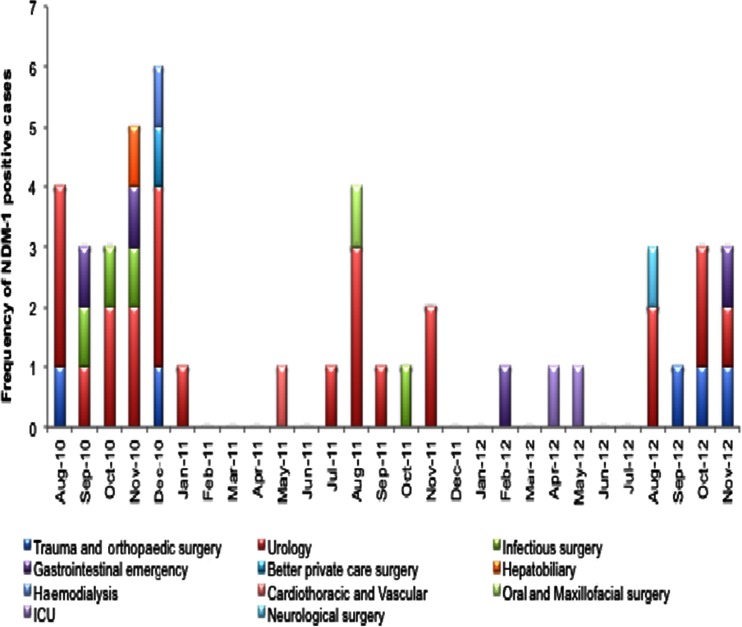
Distribution of NDM-1-positive cases by month and department (*n* = 45)

The first NDM-1-positive pathogen was *E. cloacae*, isolated from a patient in the trauma and orthopaedics department in mid-August 2010 (Fig. 2). A few days later, the second NDM-1-positive pathogen was detected in the urology department. Within a month, NDM-1-positive bacteria were detected in adjacent departments (infectious surgery, gastrointestinal emergency and hepatobiliary departments) and also in clinical departments located further away from the urology department. At the end of 2012, NDM-1-infected cases were found in 11 departments. The highest number of NDM-1-positive cases was detected in the urology department (*n* = 24), followed by the trauma and orthopaedic surgery (*n* = 5), infectious surgery (*n* = 4), gastrointestinal emergency (*n* = 4), ICU (*n* = 2) and seven other departments. Most patients were detected in November 2010 (sixcases) and December 2010 (five cases; Fig. 2). The antimicrobial susceptibilities of NDM-1-positive isolates are listed in Table 1. One bacterium, *E. cloacae*, was also resistant to colistin (>16 mg/L).

**Table 1:** Minimum inhibitory concentrations (MICs) of NDM-1-positive Enterobacteriaceae isolates (*n* = 47)

Antibiotic	Susceptible (mg/L)	Intermediate (mg/L)	Resistant (mg/L)
Imipenem	6 (0.5–1)	13 (2)	28 (>4)
Meropenem	8 (0.5–1)	5 (2)	34 (>4)
Cefotaxime	0	0	47 (>32)
Ceftazidime	0	3 (8)	44 (>32)
Ciprofloxacin	1 (<1)	2 (2)	44 (8–256)
Amikacin	14 (<16)	7 (>16)	26 (>64)
Gentamicin	2 (0.25–1)	3 (>4)	48 (>32)
Colistin	46 (0.25–1)	0	1 (>16)

Phenotypic testing for MBL of 47 NDM-1-positive isolates revealed that 42 isolates were positive. 5/47 isolates co-produced OXA-48 carbapenemase, a member of the serine beta-lactamases, whose activities are not inhibited by EDTA and, as a result, are MBL test-negative. We detected the following other carbapenem resistance genes in NDM-1-negative CRE isolates: OXA-48 (*n* = 6, *K. pneumoniae*) and KPC (*n* = 1, *E. coli*). In the remaining 14 CRE isolates, no carbapenemase genes were detected with our testing panel. Most of these CRE also carried other resistance genes conferring ESBL (Table 2).

**Table 2:** Beta-lactamase resistance genes present on plasmids of NDM-1-positive Enterobacteriaceae isolates (*n* = 47)

Species	TEM	SHV	CTX-M
*Enterobacter cloacae* (*n* = 15)	15	4	6
*Klebsiella pneumoniae* (*n* = 9)	8	ND	6
*Escherichia coli* (*n* = 12)	11	3	7
*Citrobacter freundii* (*n* = 8)	8	–	3
*Providencia rettgeri* (*n* = 1)	1	–	1
*Enterobacter aerogenes* (*n* = 1)	–	–	1
*Klebsiella oxytoca* (*n* = 1)	1	–	1
Total	44	12	25

*ND* not done, as SHV was expected to be present on the chromosome of most *Klebsiella* species

### Characterisation of NDM-1-positive bacteria

The majority of the PFGE patterns of NDM-1-positive bacteria showed that these were different, suggesting limited clonal expansion. Just three *E. cloacae* isolates and two *E. coli* isolates from the urinary tract belonged to a single PFGE profile, suggesting clonal spread.

**Fig. 3:**
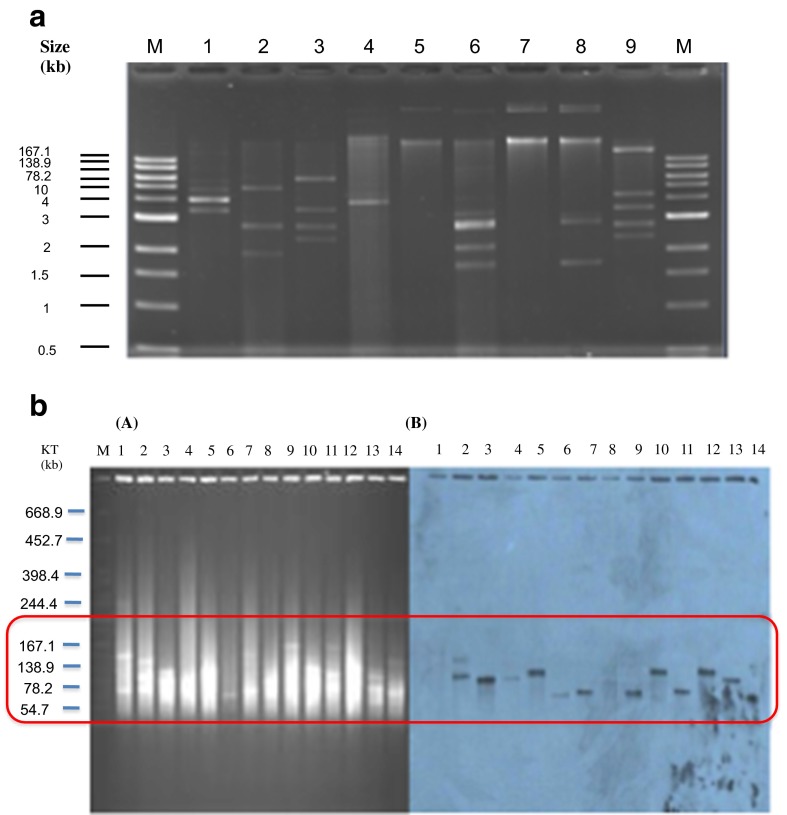
**a** The differences in plasmid number from a selection of bacteria isolates. Several isolates contained up to eight plasmids **b** S1-PFGE and Southern blotting of plasmids carrying NDM-1 from clinical isolates. Pulsed-field gel of S1-treated plasmid DNA of selected Enterobacteriaceae from clinical isolates stained with ethidium bromide. The molecular weight marker is *S. braenderup* H9812 cut with XbaI (*A*). Autodiagram of gel A showing plasmids carrying NDM-1 (*B*)

The isolates were analysed for location of the NDM-1 gene by Southern blotting, which showed that most NDM-1-positive isolates carried only one type of NDM-1 plasmid. One *E. coli* strain isolated in 2010 contained two different plasmids with the NDM-1 gene (Fig. 3). The size of the NDM-1 isolate containing plasmids ranged from 50 to 150 kb and were characterised as follows: incompatibility type FIIs-repA (*n* = 16), N-repA (*n* = 3) and IncA/C (*n* = 4). The remaining isolates could not be typed (Table 3). We found two isolates, *E. coli* and *E. cloacae*, that carried the NDM-1 gene on their chromosomes. Transconjugants were performed for 45 clinical isolates. 17.8 % (8/45) of isolates transferred their NDM-1 plasmid to *E. coli* J53. All of transconjugants were confirmed to be NDM-1-positive.

**Table 3:** Plasmid replicon types of NDM-1-positive isolates

Species	Plasmid replicon type		
	FIIs-repA	IncA/C	Untypeable
*Enterobacter cloacae* (*n* = 14)	7	1	6
*Klebsiella pneumoniae* (*n* = 9)	3	2	4
*Escherichia coli* (*n* = 11)	4	–	7
*Citrobacter freundii* (*n* = 8)	1	1	6
*Providencia rettgeri* (*n* = 1)	–	–	1
*Enterobacter aerogenes* (*n* = 1)	–	–	1
*Klebsiella oxytoca* (*n* = 1)	1	–	–
Total (*n* = 45)	16	4	25

### NDM-1 rectal carriage and presence in the environment

We screened the stool specimens of 103 patients at admission and discharge. We found five patients with NDM-1-positive bacteria at admission: *E. coli*, *K. pneumoniae*, *E. cloacae*, *P. mirabilis* and *Acinetobacter* spp. at admission. Two of the patients—both hepatobiliary surgery—were NDM-1-negative at discharge, one after spending 21 days in the hospital and the other after spending 3 days in the hospital. One patient was negative at admission and positive with *P. mirabilis* carrying the NDM-1 gene at discharge.

Screening environmental hospital samples (*n* = 200) for CRE showed the following: 138 were negative, 62 were positive of which, 31 were *Acinetobacter species*, seven *Enterobacter* species, eight *Klebsiella* species, three *E. coli*, 12 *Pseudomonas* species and a single case of *Citrobacter* species. From the 62 positive screen samples, only five were confirmed to be NDM-1-positive. Three of the five NDM-1-positive strains were *Acinetobacter* species that were isolated from the patients’ beds. The other two positive cases were *E. aerogenes*, isolated from a toilet seat and a medical waste bin. We could only classify one plasmid of the *E. aerogenes* isolate that was found on the toilet seat in the urology department. It was an N-repA plasmid, which was also found in clinical *Acinetobacter* isolates.

## Discussion

The present study demonstrates that carbapenem resistance among Enterobacteriaceae due to NDM-1 is frequently isolated in a major surgical hospital in Vietnam. NDM-1 constituted 1.1 % of all Enterobacteriaceae in our study and 68.1 % of CRE. This is comparable to the 1.2 % NDM-1 prevalence amongst Enterobacteriaceae reported by Kumarasamy and colleagues in India in 2010 [23]. We may have underestimated the number of CRE as we used a relatively high concentration of imipenem in our screening broth. In the United States, only seven NDM-1-producing CRE have been reported to the Centers for Disease Control and Prevention (CDC) from clinical isolates between 2009 and 2011, with most patients having links to travel to Asia [24]. Having found 68.1 % of all CRE to be NDM-1-positive is alarming. Furthermore, we found CRE containing OXA-48 and KPC. Still, a number of CRE isolates were negative for all the tested genes, and are currently being characterised further using sequencing techniques.

The most common bacterial species carrying NDM-1 were *E. cloacae*, *E. coli*, *K. pneumoniae* and *C. freundii*. All of them belong to the human gut flora and are widespread in the environment. NDM-1 has been detected in a river in Hanoi, illustrating the environmental spread of NDM-1 in Hanoi [12]. We found five patients who carried NDM-1 rectally at admission, demonstrating the scope of NDM-1 spread among admitted patients from the community and also the transmission of NDM-1 between different hospitals; three of the five positive patients at admission had been transferred from another hospital. An investigation of NDM-1 resistance in the other hospitals would help to better understand the resistance spread. Furthermore, in one household member of an NDM-1-positive patient, we found an NDM-1-positive *Proteus mirabilis*, again illustrating the potential spread in the community. In a recent cross-sectional community-based study, NDM-1-positive *K. pneumoniae* were cultured from the nasopharynx in two healthy volunteers in Hanoi [13]. Our examination of the hospital’s environment for NDM-1 contamination revealed three NDM-1-positive *Acinetobacter* isolates and only 6/436 carbapenem-resistant *Acinetobacter* isolates were NDM-1-positive. Possibly, *Acinetobacter* species play a role as an environmental NDM-1 reservoir that can function as a conduit of NDM-1 containing plasmids to other bacterial genera, such as *K. pneumoniae* [25, 26]. Replicate typing revealed that clinical isolates and environmental isolates contain similar plasmid types. However, plasmid genome sequencing could provide better insight into this, which was not performed.

The majority of positive cases originated from the urology department. Since the faecal flora can serve as an antibiotic resistance reservoir for uropathogens and due to the fact that most patients were catheterised may explain this finding [27]. Also, these patients may have been cultured more frequently, leading to selection bias. NDM-1-positive bacteria were detected sooner in departments adjacent to the urology department as compared to more distant departments. These observations suggest that the urology department is a potential NDM-1 hotspot from where NDM-1-positive bacteria disseminated to different departments in the hospital, either by transferred patients or by healthcare workers. In an outbreak of carbapenem-resistant *K. pneumoniae* with 18 patients, they identified three independent transmissions from a single patient that had been discharged before the second case was detected.

In our study, PFGE showed genetic diversity among NDM-1 and, thus, there is limited evidence of clonal expansion, confirming that horizontal gene transfer of NDM-1 containing plasmids is the main mechanism of spread. Besides the large variety in NDM-1-positive species, the NDM-1 gene was also carried by various plasmid types: IncA/C, IncF, IncN or untypeable, similar to other studies [28]. In the literature, the NDM-1 gene has been more frequently reported on broad host range IncA/C-type plasmids [29]. In Vietnam, most plasmids were IncFII, which has been found in Poland as well, and was recently sequenced [29–31]. The observation that the majority of NDM-1 was found on IncFII plasmids is alerting, as this plasmid type can spread efficiently, as was seen with the worldwide dissemination of *bla*_CTX-—15_-borne IncFII plasmids [29]. IncFII-type plasmids are considered narrow host range plasmids usually identified among *E. coli* strains [32]. However, in our study, IncFII were also detected in *E. cloacae*, *K. pneumoniae* and *C. freundii*. As these plasmids are characterised by several toxin–antitoxin addiction systems, they are efficiently passed on during bacterial cell division. The *Acinetobacter* isolates appear to have generally N-repA plasmids, which were not observed in Enterobacteriaceae (data not shown).

We were unable to assess the clinical significance of NDM-1 regarding antibiotic treatment and outcome, which is a limitation of our study. In a previous report from two patients in the same hospital, there was no evidence of poor clinical outcome and the NDM-1-positive bacteria were likely colonisers rather than pathogens. However, as the plasmids can be easily transferred, the potential of NDM-1 spreading to disease-causing bacteria is a serious threat and can result in high morbidity and mortality, as was seen in the UK [21]. Future studies would need to incorporate clinical data in order to obtain the full picture. Unfortunately, we did not have access to these data.

## Conclusion

With the present study, we have gained insight into the spread of an important antibiotic-resistant gene in a major hospital in Hanoi, Vietnam. We also documented patients admitted and discharged while being colonised with New Delhi metallo-beta-lactamase 1 (NDM-1)-positive bacteria and even demonstrated a household to be contaminated with NDM-1-positive bacteria. Furthermore, NDM-1 was found in environmental bacteria. This indicates that NDM-1 has infiltrated both the healthcare as well as the community settings. The issue of carbapenem resistance needs both hospital- and community-based control efforts.
